# Influence of Self-Efficacy and Motivation to Follow a Healthy Diet on Life Satisfaction of Patients with Cardiovascular Disease: A Longitudinal Study

**DOI:** 10.3390/nu12071903

**Published:** 2020-06-27

**Authors:** Rosario Castillo-Mayén, Cristina Cano-Espejo, Bárbara Luque, Esther Cuadrado, Tamara Gutiérrez-Domingo, Alicia Arenas, Sebastián J. Rubio, Javier Delgado-Lista, Pablo Pérez-Martínez, Carmen Tabernero

**Affiliations:** 1 Maimonides Biomedical Research Institute of Cordoba (IMIBIC), 14004 Córdoba, Spain; esther.cuadrado@uco.es (E.C.); tamara.gutierrez@uco.es (T.G.-D.); aarenas@us.es (A.A.); sjrubio@uco.es (S.J.R.); delgadolista@gmail.com (J.D.-L.); pabloperez@uco.es (P.P.-M.); 2 Department of Psychology, University of Cordoba, 14071 Córdoba, Spain; cristinacanoes@gmail.com; 3 Department of Psychology, University of Seville, 41018 Seville, Spain; 4 Department of Didactics of Experimental Sciences, University of Cordoba, 14071 Cordoba, Spain; 5 Lipids and Atherosclerosis Unit, Department of Internal Medicine, Reina Sofia University Hospital, 14004 Córdoba, Spain; 6 Department of Medicine (Medicine, Dermatology and Otorhinolaryngology), University of Cordoba, 14004 Cordoba, Spain; 7 CIBER Fisiopatología de la Obesidad y Nutrición (CIBEROBN), Instituto de Salud Carlos III (ISCIII), 28007 Madrid, Spain; 8 Instituto de Neurociencias de Castilla y León (INCYL), University of Salamanca, 37007 Salamanca, Spain

**Keywords:** healthy diet, cardiovascular disease, self-efficacy, motivation, life satisfaction

## Abstract

Today, cardiovascular disease has a great impact on the global population due to its high prevalence. One challenge that cardiovascular patients face to achieve a better prognosis is to follow a healthy diet. This study focused on psychological factors linked to adaptation to a healthy diet in these patients. The main objective was to analyze the interrelationship between motivation to follow a healthy diet and self-efficacy to adhere to the Mediterranean diet with life satisfaction over time. The sample consisted of cardiovascular patients who were assessed at three measurement moments (NT1 = 755; NT2 = 593; NT3 = 323, average interval time: nine months). Correlation analyses showed that self-efficacy, motivation, and life satisfaction followed a pattern of positive relations across the three measurements. A time effect over the study variables was also observed. The results of path analyses showed that self-efficacy positively predicted autonomous motivation, which in turn was associated with patients’ life satisfaction. This interrelation was stable over a period of 18 months. Moreover, life satisfaction predicted self-efficacy nine months later. Psychological interventions might be a positive resource for cardiovascular patients, since psychological variables influence their life satisfaction and their subsequent quality of life in their new health condition.

## 1. Introduction

Currently, cardiovascular disease (CVD) is receiving significant scholarly attention both due to its prevalence and its impact on the population. Specifically, CVD is the leading cause of death worldwide [1]. Thus, one major concern of health professionals internationally is to promote quality of life and life satisfaction of CVD patients when dealing with their new health condition. The appearance of this disease commonly implies the adoption and maintenance of healthy habits in relation to diet, physical exercise, or emotional regulation among others [2,3]. Good adherence to a healthier lifestyle is a predictor for cardiac risk reduction [4,5,6,7].

It is also acknowledged that there are psychological factors related to the onset, course, and prognosis of the disease [1]. Moreover, some of these factors are involved in the adherence to and maintenance of behavioral changes needed for disease adaptation [8,9,10], which in turn are especially relevant to a better disease course and prognosis. Likewise, the need for a social psychological perspective has been highlighted when it comes to understanding food choice and nutrition related-behavior [11]. In this study, given the importance of following a healthy diet for these patients, we explored the role of some psychological factors in patients’ psychological well-being. Specifically, in this study we analyzed how self-efficacy for adherence and motivation to follow a healthy diet may influence CVD patients’ life satisfaction over time.

### 1.1. Diet and CVD

Eating patterns are one of the main changes that CVD patients must introduce to achieve a good health status [3]. First, the eating habits that the patient has had until the moment of diagnosis must be studied, in order to preserve any healthy habits that they may have had so far and replace the unhealthy habits with others that are beneficial to their health. This process of a change in diet mainly requires that the patients have at their disposal the necessary information about their diet and their health; a team of professionals whom they can consult about their questions and difficulties that arise as they implement those changes in their daily routine, and a context that facilitates those implementations [12]. In addition, it requires that they can count on an intervention by health professionals in psychology to work together on psychological aspects, such as motivation, self-efficacy, or emotion regulation, among others, that can favor the required change and maintenance of healthy behaviors [2].

Given that there has been a great deal of debate regarding diet and the types of foods included in the healthy and unhealthy lists, it is more common to focus on a certain consumption pattern, rather than on individual foods. The Mediterranean diet has received the greatest recognition so far as being beneficial for cardiovascular health [13,14]. For example, the PREDIMED study [15] analyzed whether the characteristics of the Mediterranean diet can be a method of CVD prevention. Initially, 7447 people participated in this study and a follow-up was carried out for 4.8 years. Overall, the findings supported the influence of the Mediterranean diet supplemented with extra virgin olive oil or with nuts on reducing the risk of future cardiac events. In a randomized clinical trial [16], following a Mediterranean diet was shown to decrease the metabolic process involved in type 2 diabetes, called postprandial lipidemia, which is directly related to cardiovascular risk. Therefore, the Mediterranean diet is considered an appropriate intervention for CVD prevention.

### 1.2. Self-Efficacy, Motivation, Life Satisfaction, and CVD

Self-efficacy and autonomous motivation are core psychological factors involved in health behavior initiation and maintenance [17]. As for self-efficacy, Bandura [18] defined this variable as the perception that a person has about his or her own ability to perform an action to achieve a goal or objective. Social cognitive theory (SCT) postulates that the perception of self-efficacy is able to motivate behavior engagement [18]. Numerous investigations have demonstrated the central role that self-efficacy plays in individual involvement in the performance of behaviors related to health. Among them, with a focus on the CVD population, research has outlined a positive influence of self-efficacy to perform dietary changes [10,19] or for adhering to a healthy diet [20,21]. 

Along with self-efficacy, one of the most important psychological factors to execute lifestyle changes is motivation. According to the self-determination theory (SDT) [22,23], motivation can acquire different levels of internalization and can be classified as a passive or active execution. Thus, this theory distinguishes autonomous motivation, controlled motivation, and amotivation. Controlled motivation is in turn subdivided, into external regulation and introjection, while autonomous motivation is divided into identified and integrated regulations. The applicability of this theory to health behavior has been widely analyzed [24,25]. For example, in the CVD population, the relationship between this theory and smoking cessation was demonstrated in patients who had suffered a cardiovascular incident [9]. Specifically, it was found that people showed a greater tendency to act when their motivation was autonomously regulated, that is, to perform behaviors or changes in habits related to their health. These results were also significantly related to an improved physical and psychological well-being in these patients.

To unify the studies that have been based on the self-determination theory in the health context, a meta-analysis was performed to examine the relationship between the components of the theory and other aspects related to the physical and psychological health of patients [26]. In this study, the authors concluded that autonomous self-regulation is the component that exerts the greatest influence on a high level of overall patient well-being. Another finding was that long-term introjected regulation was associated with anxiety-depressive symptoms, despite the adherence to the change that it could imply at first. Therefore, in general, the results of this meta-analysis showed the benefit of self-regulated behavior in comparison to the detriment of externally controlled regulation in the maintenance of health behavior.

In addition, the joint contribution of self-efficacy and autonomous motivation has been tested in the CVD population with regards to adherence to exercise [27,28] and healthy eating [8]. The mutual influence of these two variables has been explored, with results indicating that both routes—that is, self-efficacy influencing self-determined motivation and self-determined motivation influencing self-efficacy—possibly explain exercise behavior initiation [28], but that the latter seems to be superior for predicting exercise maintenance [27] and the following of a healthy diet in diagnosed patients [8]. However, when it comes to predicting life satisfaction, it has been demonstrated that the association between this variable and autonomous self-regulation is higher than its association with self-efficacy, at least in other chronic health conditions such as diabetes [29]. 

Regarding CVD patients’ life satisfaction, previous research has concluded that it is lower when there is not adherence to the necessary changes for their health after illness [30]. As for diet-related behavior, recent studies have shown that adherence to the Mediterranean diet is positively related to quality of life [31] and life satisfaction in adults [32]. Additionally, healthy diet consumption is a relevant predictor of patients’ life satisfaction six months later, as is life satisfaction at baseline to predict a measurement 12 months later [8].

The present research sought to add more evidence on the longitudinal interrelation among self-efficacy, motivation, and life satisfaction in CVD patients by assessing their association over three measurement moments that comprised approximately 18 months overall. This would suppose a longer period of time than previously tested. Also, these variables were assessed in relation to a specific behavior, that is, the following of a healthy diet. Moreover, in terms of self-efficacy, this study focused on the adherence to the Mediterranean diet, which is a specific healthy food pattern. This study also aimed to examine the role of these psychological variables that facilitate diet adherence on CVD patients’ life satisfaction over time, as well as the potential positive effect of time over them. Specifically, according to theoretical and empirical evidence, a positive relationship was expected among the study variables over time, and that self-efficacy for adhering to a healthy diet would trigger autonomous motivation to follow such a diet, which in turn would influence patients’ life satisfaction. Thus, the study hypotheses were:

**Hypothesis** **1.** *Motivation, self-efficacy, and life satisfaction will be positively correlated in the three measurement moments, both in cross-sectional terms (Hypothesis 1a) and longitudinally (Hypothesis 1b). Thus, CVD patients who show a higher satisfaction with their lives would also show higher levels of self-efficacy to adhere to the Mediterranean diet and will be more internally motivated to follow a healthy diet.*

**Hypothesis** **2.** *There will be a positive effect of time over self-efficacy (Hypothesis 2a), autonomous motivation (Hypothesis 2b) and life satisfaction (Hypothesis 2c), so that levels of the study variables at Time 3 would be higher than baseline and Time 2 levels.*

**Hypothesis** **3.** *Baseline self-efficacy to adhere to the Mediterranean diet will positively predict self-efficacy at 9 months later (Hypothesis 3a) and 18 months later (Hypothesis 3b). This prediction will be stable over time, so that levels of self-efficacy at the second measurement moment will predict levels of this variable nine months later (Hypothesis 3c). The same pattern will be true for autonomous motivation to follow a healthy diet (Hypothesis 3d,e,f) and life satisfaction (Hypothesis 3g,h,i).*

**Hypothesis** **4.** *Baseline self-efficacy for adherence to the Mediterranean diet will positively predict baseline autonomous motivation to follow a healthy diet (Hypothesis 4a), which in turn will positively predict baseline patients’ life satisfaction (Hypothesis 4b). These associations will be stable over time, so that the same pattern will appear 9 months later (Hypothesis 4c,d) and 18 months later (Hypothesis 4e,f).*

## 2. Materials and Methods

### 2.1. Participants

This research was carried out within the framework of the CORDIOPREV study, whose characteristics have been published elsewhere [33]. All participants were patients involved in the CORDIOPREV study, who had an established coronary condition but had not suffered a clinical event in the last 6 months and had no other serious illness. The sample consisted of a total of 755 patients at Time 1 (average age = 64.60 years, SD = 9.03), in which 630 were men (83.4%) and 125 were women. At the time the data was analyzed (July, 2019), the sample at Time 2 comprised 593 patients (85.7% men) and 323 at Time 3 (88.2% men). The sociodemographic characteristics of the sample at Time 1 are shown in Table 1.

### 2.2. Instruments

Treatment Self-Regulation Questionnaire (TSRQ) [34,35]. This questionnaire was constructed from the self-determination theory [22,36] and was used to evaluate three different types of motivation to follow a healthy diet (in this study, the items related to a motivation were not considered): 6 items assessing autonomous motivation (e.g., “I follow the diet described by health professionals because I think it is the best for my health”); 4 evaluating extrinsic motivation (“I follow the diet because I want others to approve of me”); and lastly, 2 items assessing introjected motivation (“I follow the diet because I would feel guilty or ashamed of myself if I did not”). It consists of 12 items, with a 7-point scale, with 1 equaling “totally false” and 7 “totally true.”

Self-Efficacy Scale for Adherence to the Mediterranean Diet (SESAMED) [37]. This scale evaluates the self-efficacy that the patient perceives for adherence to the Mediterranean diet. This consists of 22 items, which are divided into two subscales: 12 items aimed at evaluating the self-efficacy to avoid certain foods (avoidance subscale), for example, “To what extent do you feel capable to avoid butter?”; and 10 items that assess self-efficacy for consuming other types of food (consumption subscale) that are typical of that diet, for example, “To what extent do you feel capable of consuming at least five tablespoons of olive oil per day distributed between different meals?” Each item is scored on a 7-point scale, with 1 meaning “I do not feel capable at all” and 7 being “I feel completely capable.” 

Satisfaction with Life Scale (SWLS, [38]; Spanish adaptation by [39]). This is used to assess general life satisfaction. It consists of 5 items (e.g., “I am satisfied with my life,” scored with a 7-point Likert-type scale, where 1 was “strongly disagree” and 7 was “strongly agree.” 

### 2.3. Procedure

This study was approved by the Andalusian Health Service’s Research Ethics Committee and the Reina Sofía Hospital in June 2015 (Acta 242, ref 2886, 29/06/2015). The research was carried out in the Reina Sofía Hospital in Córdoba. Participants received information related to the research beforehand, such as the purpose of the study, specifying that their participation was anonymous and voluntary. Participants gave their informed consent before participation. Three measurement moments were carried out, the first taking place in April 2016. The approximate average time between evaluations was 9 months. Participants used a tablet with Internet access on which they answered the questionnaires via the link provided for the online platform. The software used for this was QuestBack Unipark (v.10.9). During the data collection, one or two members of the research team were available to resolve possible questions.

### 2.4. Design and Data Analysis

An intragroup longitudinal design was carried out with three measurement points. Regarding statistical analyses, first, descriptive and reliability analyses were performed for the study variables. Second, Pearson’s correlations were carried out with all the study variables (global scales and subscales) in the three measurement points to analyze the relationship among them, both within the same measurement moment and in all the possible combinations among them. Third, a one-way repeated-measures ANOVA was performed with each study variable to test the effect of time in the global scores. For these analyses, SPSS software was used (v.25). Ultimately, to test the hypothesized predictive model of life satisfaction in CVD patients, path analyses were performed with AMOS software (v.22). The model was tested with the maximum likelihood method of estimation.

Model fit was evaluated using the Chi-squared statistic (χ^2^) and the Chi-square likelihood ratio (χ^2^/df). Moreover, because of the sensitivity of the χ^2^ statistic to sample size and to the violation of the multivariate normality assumption, alternative goodness-of-fit measures were employed. The descriptive measure of the overall model used was the Root Mean Square Error of Approximation (RMSEA), which assesses whether the model fits approximately well in the population. Other descriptive measures used were the Comparative Fit Index (CFI) and the Non-normed Fit Index (NNFI), since they are sensitive to model misspecification and are less sensitive to sample size than χ^2^ [40]. For model comparisons, parsimony indices considered were the RMSEA and the Akaike Information Criterion (AIC), with lower values of these indices indicating a better model fit. Additionally, the χ^2^ difference test was computed when two nested models were compared. This test examines whether both models show an equal model fit by considering the χ^2^ change (∆χ^2^), given the change in the degrees of freedom. In model building, a significant result suggests a better model fit after adding new path(s), while in model trimming the same result suggests that the model has been oversimplified [41]. For model evaluation, we followed Schermelleh-Engel, Moosbrugger and Müller’s [40] recommendations: acceptable model fit is indicated by χ^2^/df equal to or less than 3, CFI and NNFI equal to or higher than 0.95, and RMSEA lower than 0.08, with Confidence Interval (CI) close to RMSEA. Good model fit is indicated by χ^2^/df equal or less than 2, CFI and NNFI equal or higher than 0.97, and an RMSEA between 0 and 0.05, with a CI close to the RMSEA, and the left boundary of CI equal to 0.00.

## 3. Results

Descriptive data (mean and standard deviation) and Cronbach’s alpha values of all the study variables are shown in Table 2. As for correlational analyses, Table 2 also shows the results from both global scores and the subscales during the three measurement points. Overall, the results indicated a significant and positive relationship between global and subscale scores of self-efficacy and motivation with life satisfaction, with higher coefficient values of motivation than those for self-efficacy. This result was stable over time when considering relationships within the same measurement moment. The only exception to this pattern was the lack of association between self-efficacy (global and subscales) and extrinsic motivation. As for correlations of the variables between different measurement points, results indicated greater consistency and strength of the relationship between self-efficacy (global and subscales scores) with autonomous motivation; and greater consistency and strength of the relationship between global and autonomous motivation with life satisfaction. Life satisfaction scores also showed positive correlations with all the remaining study variable scores of the subsequent measurement points. 

**Figure 1:**
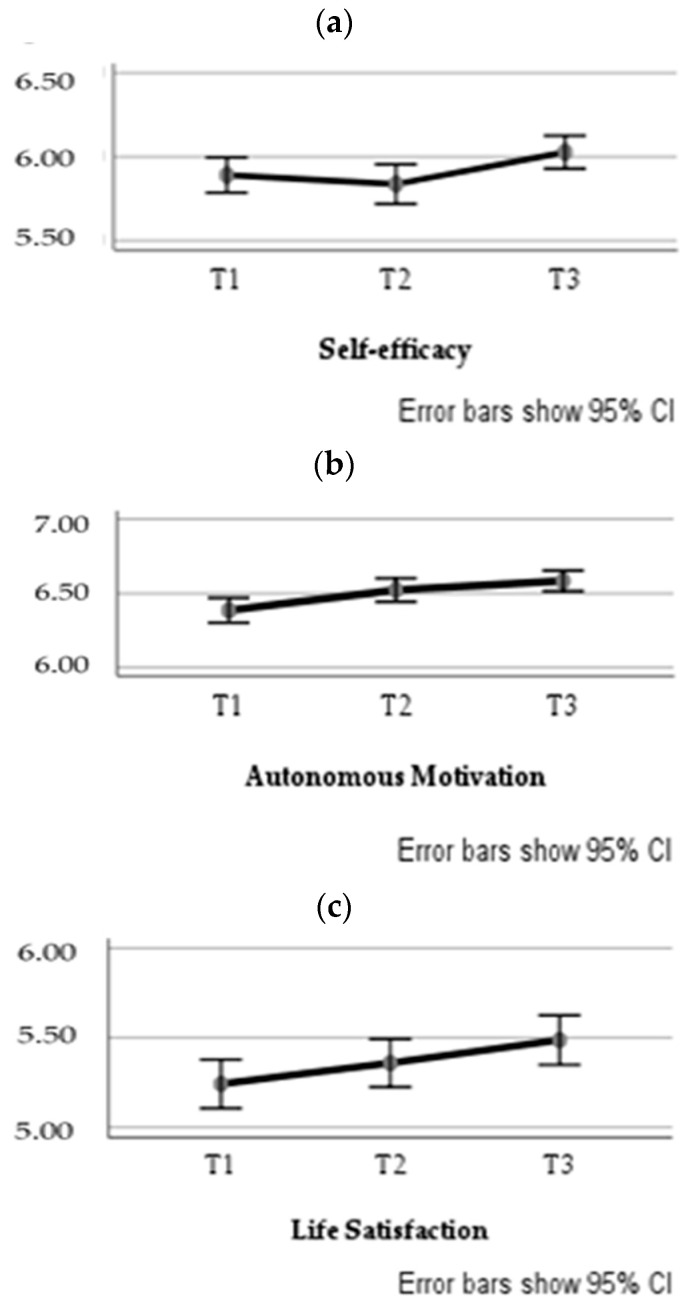
Estimated marginal means of self-efficacy (**a**), autonomous motivation (**b**), and life satisfaction (**c**) over the three measurement moments. Note. T1 = Time 1; T2 = Time 2; T3 = Time 3. CI = Confidence Interval.

A one-way repeated measures ANOVA was performed for each study variable, with the measurement moment (Time 1, Time 2, and Time 3) as the independent variable. The multivariate tests were Pillai’s Trace = 0.03, *F*(2, 321) = 5.22, *p* = 0.006, η_p_^2^ = 0.03, observed power = 0.83 for self-efficacy, Pillai’s Trace = 0.06, *F*(2, 321) = 9.64, *p* < 0.001, η_p_^2^ = 0.06, observed power = 0.98 for autonomous motivation, and Pillai’s Trace = 0.04, *F*(2, 321) = 7.45, *p* = 0.001, η_p_^2^ = 0.04, observed power = 0.94 for life satisfaction. Mauchly’s tests indicated that the assumption of sphericity had been violated when analyzing self-efficacy, χ^2^(2) = 6.58, *p* = 0.037, and autonomous motivation, χ^2^(2) = 8.80, *p* = 0.012. Since Greenhouse-Geisser’s correction was ε > 0.75 for both variables, degrees of freedom were corrected using Huynh-Feldt estimates of sphericity [42], which were ε = 0.99 for self-efficacy and ε = 0.98 for autonomous motivation. The results showed a significant main effect of time on the levels of self-efficacy, *F*(1.97, 635.03) = 4.50, *p* = 0.012, η_p_^2^ = 0.01, observed power = 0.76, autonomous motivation, *F*(1.96, 630.82) = 10.68, *p* < 0.001, η_p_^2^ = 0.03, observed power = 0.99, and life satisfaction, *F*(2, 644) = 7.45, *p* = 0.001, η_p_^2^ = 0.02, observed power = 0.94. Pairwise comparisons using Bonferroni correction indicated that self-efficacy levels at Time 2 (*M* = 5.84) significantly differed from levels at Time 3 (*M* = 6.03), *p* = 0.006; baseline levels of autonomous motivation (*M* = 6.39) significantly differed from levels at Time 2 (*M* = 6.58), *p* = 0.010, and at Time 3 (*M* = 6.58), *p* < 0.001; and life satisfaction levels at Time 1 (*M* = 5.24) significantly differed when compared with levels at Time 3 (*M* = 5.49), *p* < 0.001. Overall, results suggested a significant increase in the three study variables over time (see Figure 1).

Concerning the path analysis, we followed a multi-step process to test the contribution of each variable to predict life satisfaction levels at Time 3. This process allowed for non-nested and nested model comparisons. The results from relevant fit indices for each model are shown in Table 3 (only the final model is graphically shown in the main manuscript but all the additional models tested are also represented graphically as Supplementary Material Figures S1–S7). First, and considering the repeated measures analysis results suggesting a time effect over the variables’ levels, we started with Model 0 that tested the influence of baseline life satisfaction over Time 2 and Time 3 levels, as well as the influence of Time 2 life satisfaction over Time 3 levels. As anticipated, because of the only contribution of one variable to the model, some indices reached their maximum value (i.e., CFI), while others could not be calculated (i.e., *p*-value) or indicated a poor fit (i.e., RMSEA). We next tested this baseline model including the influence of autonomous motivation (Model 1). The new paths of this model were baseline autonomous motivation over Time 2 and Time 3 levels, Time 2 autonomous motivation over Time 3 levels, baseline autonomous motivation over baseline life satisfaction, Time 2 autonomous motivation over Time 2 life satisfaction, and Time 3 autonomous motivation over Time 3 life satisfaction. This model had an acceptable fit with the data (Table 3). Next, we tested a model in which the influence of self-efficacy for adherence to a healthy diet was considered (Model 2). This model coincided with the hypothesized model. Thus, the model included the paths: baseline self-efficacy over Time 2 and Time 3 levels, Time 2 self-efficacy over Time 3 levels, and baseline, Time 2, and Time 3 self-efficacy levels over baseline, Time 2, and Time 3 autonomous motivation levels, respectively. Although most indices indicated an acceptable fit to the data, AIC values suggested that this model was not superior to the previous one. However, given that it included all the study variables, it was decided to further explore the adequacy of the model. At this point, the significance of the individual paths was considered. All the paths hypothesized were significant except for the prediction of baseline levels of self-efficacy over Time 3 levels, which was non-significant. A nested model was then tested after removing this non-significant path (Model 3); however, the results were very similar to the previous model, but with an AIC value slightly higher than that of Model 2, suggesting a better fit of Model 2. Also, the χ^2^ difference test was calculated between Models 2 and 3, which resulted as non-significant, suggesting an equal fit for both models. Based upon theoretical implications, and in spite of its lower AIC value than that of Model 1, Model 2 was decided to be retained for further analyses. 

**Figure 2:**
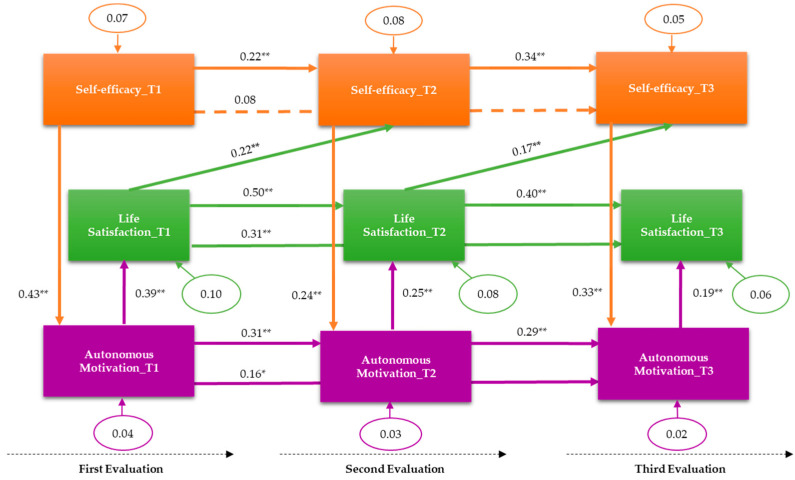
Predictive model of life satisfaction in CVD patients. Standardized coefficients and error terms (circles) are specified. ** *p* < 0.001; * *p* < 0.05. The pathway between self-efficacy at T1 and self-efficacy at T3 was non-significant (*p* > 0.05).

Additionally, although we did not make any prediction about other pathways, the correlational results suggested a potential influence of life satisfaction on self-efficacy and autonomous motivation levels of later measurement moments, so that, grounded on the Model 2, a new nested model was tested. To avoid an excess of complexity, the new paths only included the influence of life satisfaction over later measurements of self-efficacy (Model 4). The results showed that relations were significant for the link between baseline life satisfaction and self-efficacy at the second measurement point, and for life satisfaction at the second measurement point with self-efficacy at the third measurement point. A significant χ^2^ difference test comparing Models 2 and 4 suggested a better fit for the model with new paths (Model 4), so that it was further analyzed. After removing the non-significant link between baseline life satisfaction and self-efficacy at the third measurement point, the model was tested again (Model 5). As shown in Table 3, the results of Models 4 and 5 were very similar, indicating a good fit for both models, but suggesting a slight superiority of Model 5 over Model 4. The χ^2^ difference test with Model 4 resulted as non-significant, indicating equal fit, but significant when compared to Model 2, suggesting a better fit of the new model tested. Moreover, Model 5 indices indicated a better fit to the data than those of Model 2. For these reasons, Model 5 was chosen as the final model, which is represented in Figure 2. A regression analysis indicated that the final model explained 49% of the total variance of life satisfaction at T3, *R*^2^*_adj_* = 0.49, *F*(8, 314) = 39.14, *p* < 0.001.

Finally, we tested the potential influence of sociodemographic variables (measured at Time 1) on the final model. Thus, we introduced new pathways that included the links between age, socioeconomic status, and education level, with baseline, Time 2, and Time 3 life satisfaction levels (Model 6). As shown in Table 3, only some indices indicated that this model had an acceptable fit. The significant new paths were between age and life satisfaction at the three measurement moments, and between socioeconomic status and Time 3 life satisfaction, all indicating a positive effect of these variables on life satisfaction. After removing the non-significant paths, the model was tested again (Model 7). Most indices suggested that this model had a better fit than Model 6 (Table 3), but a poorer fit and a lower AIC value than Model 5.

## 4. Discussion

The objective of this study was to analyze the interrelation between motivation to follow a healthy diet, self-efficacy to adhere to the Mediterranean diet, and perceived life satisfaction of patients with CVD over time, and to ascertain the predictive ability of motivation and self-efficacy on life satisfaction.

The results allowed us to mostly confirm Hypothesis 1, in which we expected positive and significant relationships between the study variables in both cross-sectional terms (Hypothesis 1a) and longitudinally (Hypothesis 1b). Life satisfaction was significantly and positively related to all variables (global scales and subscales), in both cross-sectional terms and longitudinally, excluding only some exceptions when relating different measurement points. One of the most consistent and strong patterns of positive correlations was between global and autonomous motivation and global self-efficacy and its subscales. The lack of a relationship between global self-efficacy and extrinsic motivation (except for a negative correlation between consumption self-efficacy at T1 and extrinsic motivation at T2 and T3) was also consistent. Subscales of self-efficacy for avoidance and for consumption showed a similar pattern of correlations with respect to the motivation subscales over time to the global self-efficacy pattern. The positive associations between motivation and self-efficacy are in line with SDT and SCT approaches. Additionally, the absence of relation to external motivation might be due to the fact that—although both motivation and self-efficacy are regulatory variables of behavior—autonomous behavior is motivated by the individual’s control, in contrast to behavior that is extrinsically motivated. Extrinsic motivation depends on the consequences received from the environment for carrying out a certain behavior [22], so it is reasonable to find a weak or null association with self-efficacy, which is related to the individual’s perception of their abilities [18]. 

Another consistent result was the positive association between motivation and life satisfaction, with the stronger association being for autonomous motivation. This result is in agreement with SDT, which expects that internally motivated behavior would benefit subjective well-being [23]. This leads us to conclude, therefore, that there is stronger support for the expectation that CVD patients who are autonomously motivated to follow a healthy diet are more likely to feel more satisfied with their lives than externally motivated patients. Considering the high importance for CVD patients to maintain a healthy diet, it would be recommended for health professionals involved with these patients to facilitate this sort of motivation when interacting with them.

Concerning repeated measures analyses, the results partially confirmed Hypothesis 2 predictions. Overall, the results revealed increased levels of the three study variables over time. Specifically, Time 3 levels of the three psychological variables involved in a healthy diet were significantly higher than Time 2 levels when referring to self-efficacy and autonomous motivation and higher than baseline levels when referring to autonomous motivation and life satisfaction. The overall stability of Cronbach’s alpha values across the three measurement moments does not suggest that this increase in the study variables’ levels over time could be explained by a practice effect [43]. In addition, the interval time of nine months between each measurement point seems to be enough to prevent a practice and a carry-over effect. Thus, this time effect might be promising for adherence to a healthy diet in the CVD population. As discussed later, the self-efficacy theory postulates a mutual influence between mastery experiences in health-related behavior and self-efficacy [44], which could explain its increase over time. Also, a nutritional intervention based on SDT suggested beneficial changes in long-term dietary intakes [45]. Therefore, increased autonomous motivation to follow a healthy diet could facilitate the positive effects of this type of intervention.

Path analyses results supported that, in CVD patients: (1) previous levels of self-efficacy for adherence to the Mediterranean diet were able to predict levels of this variable nine months later, and that this prediction was stable, also occurring between a second and a third measurement point with a nine-month interval, (2) autonomous motivation levels at T1 were able to predict levels of this variable 9 and 18 months later, and autonomous motivation at T2 predicted levels of this variable at T3, and (3) baseline life satisfaction also predicted levels of this variable 9 and 18 months later, which resulted as also being stable over time since life satisfaction levels at T2 were able to predict levels of this variable at T3. These results allow us to confirm Hypothesis 3 and its derivations, except for Hypothesis 3b, which was not confirmed. These results also supported Hypothesis 4 concerning the interrelation of the study variables. Specifically, patients’ baseline levels of self-efficacy predicted their levels of autonomous motivation, which in turn predicted their life satisfaction. Moreover, this interrelationship was stable over time at three measurement points, covering a total period of 18 months. 

It is also important to note that predictions related to Hypotheses 3 and 4 were more strongly supported when additional interrelations between the study variables were included in the hypothesized model. Thus, the final model showed a better fit when it included the assumption that baseline life satisfaction would predict self-efficacy levels at T2, and the assumption that life satisfaction at T2 would predict self-efficacy levels at T3, as well. In sum, the results suggest that patients with higher levels of self-efficacy would more likely be autonomously motivated to follow a healthy diet, that autonomously motivated patients are more likely to feel more satisfied with their lives, and that patients with higher levels of life satisfaction are more likely to trust more in their ability to follow a healthy diet nine months later. Moreover, the results supported the stability of the model even after accounting for the influence of sociodemographic factors such as age and socioeconomic status that may have an impact on life satisfaction and on behavior related to cardiovascular health [30]. Also, this final model is related to findings reporting an association between lower life satisfaction and non-adherence to healthy lifestyles in CVD patients [30]. Thus, although behavior was not directly assessed in this study, given that self-efficacy and motivation are important predictors of behavior and essential factors for deliberate actions, it could be assumed that higher levels of these two variables would have led to the patient to be more likely to adhere to a healthy diet, which in turn would have enhanced life satisfaction in CVD patients. In addition, this pattern of results in which life satisfaction seems to positively influence later levels of self-efficacy resembles the so-called upward spiral of self-efficacy—for an application to health behavior see [44]—according to which mastery experiences increase self-efficacy, which, in turn, facilitate more mastery experiences. Applied to our study, mastery experiences of a healthy diet would help to increase self-efficacy for adhering to a healthy diet, which in turn would increase more opportunities to successfully perform healthy diet-related behaviors in CVD patients. In addition, according to our results, life satisfaction derived from adherence facilitates this upward spiral, influencing self-efficacy. Furthermore, this result can also be explained considering the upward spiral theory of lifestyle change [46], according to which positive affect facilitates long-term adherence to health-related behavior. The increase over time of the study variables’ levels also supports the upward spiral metaphors. Thus, both theoretical approaches can be used to explain this study’s findings, highlighting the role of self-efficacy and positive affect (i.e., life satisfaction) in promoting adherence to a healthy diet. Future studies should include a direct assessment of healthy diet-related behavior to adequately test these theoretical assumptions.

Overall, this study’s results are in line with previous research on the CVD population about the positive influence of self-efficacy on diet-related behavior [10,20], the role of motivation in other relevant health behaviors [9], as well as the joint contribution of these two variables to adherence to exercise [27,28] and healthy eating [8]. However, the interrelation of motivation and self-efficacy in the present study differs from that proposed in previous research when it comes to explaining healthy behavior, where self-determined motivation preceded self-efficacy. In contrast, our results are in line with findings from patients with another chronic condition (diabetes) in which both variables were linked to life satisfaction, but the association with autonomous regulation was stronger [29]. Thus, it could be suggested that motivation would precede self-efficacy, which in turn would be closer to health behavior attainment, while self-efficacy would precede autonomous motivation in affective facets such as life satisfaction. Also, the fact that autonomous motivation is more proximal to life satisfaction is coherent with the idea that this type of motivation is intimately linked to the individuals’ personal values [22,23]. 

Taken together, these results indicate that promoting confidence in one’s ability and autonomous motivation to follow a healthy diet make it easier for CVD patients to sustain more favorable life satisfaction over time. Patients that succeed in integrating following a healthy diet as a personal value and in attaining confidence in their ability to adhere to a healthy diet, such as the Mediterranean diet, are more likely to obtain greater positive well-being and, thus, better adjustment to their new health condition. These results are relevant considering that for these patients, it is very important to stick to a healthy diet. Thus, one important practical implication of these findings is the psychological intervention to develop and maintain self-efficacy and autonomous motivation to follow the dietary recommendations, which would probably help CVD patients to achieve two important goals: to maintain, or improve if necessary, both their physical and their psychological health status. 

More specifically, for psychology professionals, the results obtained in this research have several practical applications. The influence of psychological factors that have to do with the self-regulation of diet behavior, such as motivation and self-efficacy, in the life satisfaction of people diagnosed with CVD suggests intervention approaches. Therefore, it is important that psychological interventions are more available to this population and that they favor the development of these factors to contribute to those patients having an adequate quality of life, despite their physical condition. As indicated by the American Heart Association [47], scientific evidence suggests that self-efficacy enhancement is a therapeutic strategy that promotes behavior change, including physical activity and healthy eating. Thus, a psychological intervention aimed to promote adherence to a healthy diet and sustained internal motivation would benefit from considering Bandura’s SCT [18,48,49]. According to this theoretical approach, there are four central sources that facilitate the development of self-efficacy beliefs [48] that might be implemented across a CVD self-management program [50]: mastery experiences, vicarious, or indirect experiences, social or verbal persuasion, and physiological and affective states feedback. A pilot testing of an mHealth healthy eating program for CVD patients based on these four sources has shown initial positive results [51]. 

Thus, based on previous evidence [47,48,49,50,51,52] and in accordance with this study’s findings, we suggest that patients could enhance their motivation and long-term adherence to diet with interventions directed toward these four sources. First, it would be recommended that such interventions would start with a number of individual sessions with a focus on mastery experiences, since they have demonstrated to be the more influential source of self-efficacy [47]. In this sense, self-monitoring of healthy eating could be a central therapy resource that would facilitate the patient’s awareness of his or her own behavior capability. To increase self-efficacy from this source, a start point could be remembering past mastery experiences of healthy diet behavior. The next step would be setting goals that are reasonable and relevant to the patient’s current behavior. It is important that the person successfully achieve these goals with a certain effort, so that they should be attainable in the short term. In case of failure, it would be recommended that they persist and gain experience overcoming potential obstacles. For this process, the patients would monitor every progress in incorporating a healthy diet into their lives. Regarding the second source, group therapy could be a central component of the psychological intervention if the therapist wants to promote self-efficacy by means of vicarious or indirect experiences. In these group sessions, the patient would observe other patients being successful in adhering to a healthy diet [53]. They could also observe how others, who are similar to them, deal with difficulties and incorporate these healthy habits regardless of some obstacles. Also, they could learn and start using strategies that facilitate other CVD patients’ success. These group sessions would facilitate self-efficacy enhancement via social persuasion as well, which is the third source of self-efficacy. This consists of receiving verbal messages from relevant people who believe the person possesses the capability to succeed in the desired behavior (healthy eating). Relevant people would include the psychologist guiding the group sessions [54], the other group members, the patient’s family members, and other health professionals that have regular contact with her or him. Finally, to enhance self-efficacy via physiological and affective states feedback, it would be relevant to increase patient awareness of somatic signals and mood changes, facilitate emotional expression, and learn to manage and reduce stress reactions. Moreover, increasing a sense of self-efficacy may contribute to an improved management of physiological states [55]. It is also relevant for the patient to correctly interpret their physical states (for example, a momentary heart rate increase may have different meanings, not all of them being negative), and associate somatic and affective states with success in behavior change. For example, feeling less fatigued could be linked to weight loss [47]. Similarly, feeling more satisfied or joyful could be related to success in adhering to healthy eating. In sum, it would be expected that psychological interventions guided by SCT would help that the patients obtain a higher sense of self-efficacy to adhere to a healthy diet, that is, that they more firmly believe they have the capability to perform the desired behavior and to obtain the expected positive results. Consequently, these beliefs would facilitate the healthy behavior change and that they persist on this new habit despite any obstacles or difficulties that may alter their behavior.

Lastly, this study has certain limitations that should be noted and that at the same time can guide the design of subsequent investigations. One limitation is that we cannot determine whether the psychological variables investigated have an impact in more objective measures of adherence to a healthy diet, such as actual behavior of healthy food intake, or, even more interesting, in clinical outcomes, such as cardiovascular events. Previous studies in which these relationships were possible to be analyzed have pointed out an association between lower life satisfaction and lack of adherence to a healthy lifestyle (i.e., physical activity) [30]. Also, autonomous motivation has been related to a reduced cortisol response and to DNA methylation of the TNF gene [56]. This gene is associated with the expression of TNFα, a proinflammatory cytokine negatively associated with positive affect in patients with CVD [57]. Health motivation for food choices has been recently linked to nutritional biomarkers in adolescents [58]. Considering this and other evidence about biomarkers and psychological factors, future research should investigate whether the longitudinal model involving psychological variables related to the healthy diet proposed in this study can also be translated into better clinical outcomes in CVD patients. Another limitation is the low representation of women. In future research, it would be better to have a more balanced number of females and males, since the disease is equally important for both sexes. Third, this study was carried out in a hospital center that cares for patients in a specific geographic area, where there is a great familiarity with the Mediterranean diet. Therefore, it would be necessary to carry out similar studies in other areas in order to increase the likelihood of generalizing the results, considering other areas or countries where the Mediterranean diet is not so widespread. In such places, it would be expected that patients would have less social support to follow that diet, so that following such a diet would be less important for both the person and their environment. An extension of this study could consist of evaluating the influence of social support and identity associated with culinary culture to follow a healthy diet in these contexts, and evaluating how these variables would be associated with the life satisfaction of people with CVD. In terms of future lines of research on this topic, we also propose to examine the role of another type of variable, such as social support in CVD, as previously studied [59], or those derived from CVD risk factors, such as stress [1], in patients’ life satisfaction. Also, the proposed model could be tested in different populations with other chronic conditions that also require modifications to their daily eating routines to maintain an elevated and satisfactory life expectancy, as is the case with diabetes or Crohn’s disease, among others. 

## 5. Conclusions

This study indicates the positive interrelation between self-efficacy, autonomous motivation to follow a healthy diet, and life satisfaction in patients with CVD. Thus, promoting self-efficacy and motivation for this new lifestyle would benefit patients’ physical health status and psychological well-being. In sum, our findings highlight the importance of self-efficacy preceding motivation in order to enhance life satisfaction, the stability of this interrelation in CVD patients, and the subsequent influence of life satisfaction on self-efficacy, which completes the circle of this reciprocated influence.

## Figures and Tables

**Table 1:** Sociodemographic characteristics of the sample.

	*n*	Valid Percentage
Education level		
Basic or primary education	509	67.5
Secondary education	94	12.5
Vocational school	75	9.9
College/University	76	10.1
Employment situation		
Unemployed	59	7.8
Part-time job	25	3.3
Full-time job	145	19.2
Retired	526	69.7
Marital status		
Single	29	3.8
Living together	10	1.3
Married	635	84.2
Separated	15	2
Divorced	23	3.1
Widow/er	42	5.6
Socioeconomic status (annual income)		
<€10,800	227	30.1
€10,800–22,000	314	41.7
€22,000–43,000	114	15.1
>€43,000	25	3.3
I prefer not to answer	73	9.7

**Table 2:** Correlations, means (M), standard deviations (SD), and Cronbach’s alpha values of global scales and subscales at the three measurement moments.

	T1								T2								T3							
	1	2	3	4	5	6	7	8	1	2	3	4	5	6	7	8	1	2	3	4	5	6	7	8
**Time 1** 1. Gl. self-efficacy		0.91 **	0.77 **	0.16 **	0.32 **	−0.04	0.13 **	0.17 **	0.31 **	0.26 **	0.26 **	0.04	0.14 **	−0.07	0.07	0.09 *	0.21 **	0.20 **	0.13 **	0.07	0.18 **	−0.06	0.13 *	0.13 *
2. Avoid. self-efficacy			0.43 **	0.14 **	0.25 **	−0.01	0.11 **	0.13 **	0.28 **	0.29 **	0.14 **	0.06	0.12 **	−0.02	0.07	0.04	0.15 **	0.19 **	0.04	0.08	0.15 **	−0.02	0.10 *	0.10 *
3. Cons. self-efficacy				0.13 **	0.31 **	−0.06	0.11 **	0.17 **	0.25 **	0.11 **	0.37 **	−0.02	0.11 **	−0.13 **	0.03	0.13 **	0.22 **	0.15 **	0.24 **	0.03	0.16 **	−0.12 *	0.11 *	0.13 *
4. Gl. motivation					0.64 **	0.78 **	0.73 **	0.30 **	0.12 **	0.13 **	0.04	0.37 **	0.26 **	0.25 **	0.35 **	0.23 **	0.13 *	0.16 **	0.04	0.42 **	0.29 **	0.33 **	0.29 **	0.27 **
5. Aut. motivation						0.10 **	0.42 **	0.28 **	0.20 **	0.16 **	0.18 **	0.22 **	0.35 **	−0.01	0.26 **	0.20 **	0.24 **	0.23 **	0.14 **	0.22 **	0.36 **	0.01	0.22 **	0.28 **
6. Ext. motivation							0.36 **	0.20 **	−0.00	0.05	−0.09 *	0.30 **	0.06	0.35 **	0.20 **	0.17 **	−0.03	0.00	−0.07	0.35 **	0.09	0.41 **	0.19 **	0.15 **
7. Int. motivation								0.16 **	0.10 *	0.10 *	0.06	0.26 **	0.20 **	0.11 **	0.33 **	0.11 *	0.15 **	0.16 **	0.07	0.30 **	0.24 **	0.21 **	0.24 **	0.17 **
8. Life satisfaction									0.22 **	0.18 **	0.18 **	0.21 **	0.18 **	0.12 **	0.19 **	0.60 **	0.21 **	0.21 **	0.13 *	0.24 **	0.26 **	0.13 *	0.18 **	0.61 **
**Time 2** 1. Gl. self-efficacy										0.91 **	0.72 **	0.22 **	0.40 **	−0.01	0.21 **	0.18 **	0.39 **	0.37 **	0.28 **	0.14 **	0.23 **	0.02	0.13 *	0.18 **
2. Avoid. self-efficacy											0.36 **	0.22 **	0.37 **	0.00	0.21 **	0.14 **	0.36 **	0.40 **	0.15 **	0.16 **	0.22 **	0.06	0.12 *	0.17 **
3. Cons. self-efficacy												0.13 **	0.27 **	−0.03	0.12 **	0.17 **	0.29 **	0.17 **	0.38 **	0.05	0.16 **	−0.06	0.09	0.13 **
4. Gl. motivation													0.63 **	0.81 **	0.76 **	0.31 **	0.14 **	0.17 **	0.03	0.50 **	0.35 **	0.40 **	0.36 **	0.22 **
5. Aut. motivation														0.13 **	0.43 **	0.29 **	0.27 **	0.28 **	0.16 **	0.29 **	0.45 **	0.08	0.20 **	0.25 **
6. Ext. motivation															0.42 **	0.18 **	−0.04	−0.00	−0.08	0.41 **	0.11 *	0.45 **	0.28 **	0.08
7. Int. motivation																0.25 **	0.14 **	0.17 **	0.04	0.40 **	0.31 **	0.27 **	0.33 **	0.21 **
8. Life satisfaction																	0.22 **	0.22 **	0.13 **	0.21 **	0.24 **	0.11 **	0.14 **	0.65 **
**Time 3** 1. Gl. self-efficacy																		0.91 **	0.74 **	0.31 **	0.47 **	0.07	0.26 **	0.26 **
2. Avoid. self-efficacy																			0.40 **	0.30 **	0.43 **	0.08	0.26 **	0.23 **
3. Cons. self-efficacy																				0.21 **	0.35 **	0.03	0.17 **	0.20 **
4. Gl. motivation																					0.63 **	0.82 **	0.74 **	0.33 **
5. Aut. motivation																						0.18 **	0.42 **	0.35 **
6. Ext. motivation																							0.40 **	0.18 **
7. Int. motivation																								0.24 **
*M*	50.88	50.98	50.76	50.39	60.39	30.87	50.41	50.11	50.89	60.01	50.72	50.59	60.52	40.16	50.67	50.26	60.03	60.16	50.88	50.60	60.57	40.08	50.71	50.47
*SD*	00.99	10.29	10.01	00.90	00.79	10.68	10.75	10.36	10.04	10.42	10.03	00.91	00.74	10.68	10.76	10.36	00.88	10.19	00.87	00.87	00.66	10.61	10.74	10.27
Cronbach’s Alpha	0.93	0.96	0.83	0.79	0.89	0.76	0.87	0.86	0.94	0.94	0.81	0.79	0.87	0.74	0.89	0.86	0.92	0.96	0.77	0.78	0.86	0.70	0.86	0.85

Note. T1: Time 1; T2: Time 2; T3: Time 3; Gl.: Global; Consump.: Consumption; Aut.: Autonomous; Ext.: Extrinsic; Int.: Introjected; M: Mean; SD: Standard Deviation. * *p* < 0.05, ** *p* < 0.01.

**Table 3:** General theoretical model—fit indices of path models following a multi-step process to test the longitudinal contribution of each variable on life satisfaction at Time 3.

Model	χ2 (df)	*p*	χ^2^/df	CFI	NNFI	RMSEA (90% CI)	AIC	∆χ^2^ (df)/*p*
Model 0	0.00 (0)	--	--	1.00	--	0.565 (0.513–0.619)	12	-
Model 1	14.86 (6)	0.021	2.48	0.98	0.96	0.068 (0.024–0.112)	44.86	-
Model 2	51.81 (21)	<0.001	2.47	0.96	0.93	0.067 (0.045–0.091)	99.81	-
Model 3	54.26 (22)	<0.001	2.47	0.96	0.93	0.067 (0.045–0.090)	100.26	M2 → M3: χ^2^(1) = 2.45/.118
Model 4	24.73 (18)	0.113	1.37	0.99	0.98	0.034 (0.000–0.064)	78.73	M2 → M4: χ^2^(3) = 27.08/< 0.001
**Model 5**	**24.87 (19)**	**0.165**	**1.31**	**0.99**	**0.99**	**0.031 (0.000–0.061)**	**76.87**	**M4 → M5: χ^2^(1) = 0.14/.708** **M2 → M5: χ^2^(2) = 26.94/<0.001**
Model 6	119.77 (40)	<0.001	2.99	0.91	0.85	0.079 (0.063–0.095)	195.77	-
Model 7	59.18 (34)	0.005	1.74	0.97	0.95	0.048 (0.026–0.068)	123.18	-

Note. CFI = Comparative Fit Index; NNFI = Non-normed Fit Index; RMSEA = Root Mean Square Error of Approximation; CI = Confidence Interval; AIC = Akaike Information Criterion. ∆χ^2^ = χ^2^ change. Results from the final model are in bold.

## Supplementary Materials

The following are available online at https://www.mdpi.com/2072-6643/12/7/1903/s1, Figures S1–S7. Figure S1. Model 0: Predictive model of life satisfaction in CVD patients considering only life satisfaction over time. Figure S2. Model 1: Predictive model of life satisfaction in CVD patients considering only life satisfaction and autonomous motivation over time. Figure S3. Model 2: Predictive model of life satisfaction in CVD patients considering life satisfaction, autonomous motivation and self-efficacy over time. Figure S4. Model 3: Predictive model of life satisfaction in CVD patients considering life satisfaction, autonomous motivation and self-efficacy over time. Figure S5. Model 4: Predictive model of life satisfaction in CVD patients considering life satisfaction, autonomous motivation and self-efficacy over time, and the influence of life satisfaction over later measurements of self-efficacy. Figure S6. Model 6: Predictive model of life satisfaction in CVD patients considering life satisfaction, autonomous motivation and self-efficacy over time, the influence of life satisfaction over later measurements of self-efficacy, and the influence of sociodemographic variables. Figure S7. Model 7: Predictive model of life satisfaction in CVD patients considering life satisfaction, autonomous motivation and self-efficacy over time, the influence of life satisfaction over later measurements of self-efficacy, and the influence of sociodemographic variables.
