# Supplementary material for: Influence of Self-Efficacy and Motivation to Follow a Healthy Diet on Life Satisfaction of Patients with Cardiovascular Disease: A Longitudinal Study
Source: Nutrients. 2020 Jun 27;12(7):1903. doi: 10.3390/nu12071903 (PMC7400119; doi:10.3390/nu12071903)
Supplement: Supplementary file 1 [file nutrients-12-01903-s001.pdf]

nutrients

Supplementary Materials:

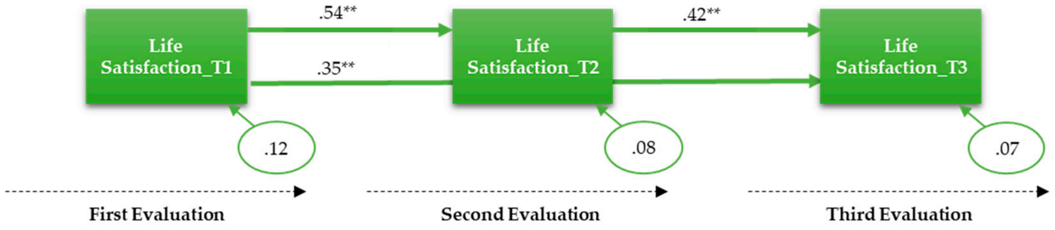

Figure S1. Model 0: Predictive model of life satisfaction in CVD patients considering only life satisfaction over time. Standardized coefficients and error terms (circles) are specified. ** $p < .001$.

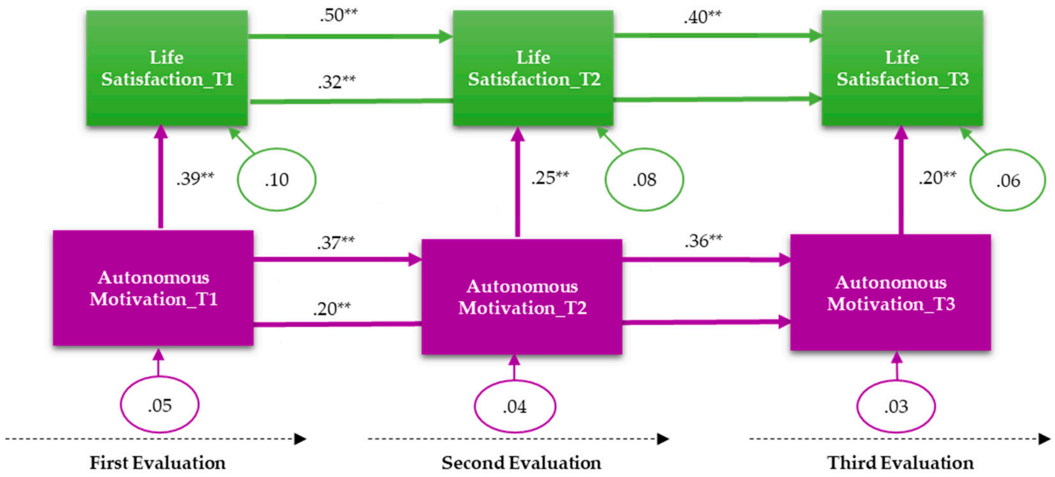

Figure S2. Model 1: Predictive model of life satisfaction in CVD patients considering only life satisfaction and autonomous motivation over time. Standardized coefficients and error terms (circles) are specified. ** $p < .001$.

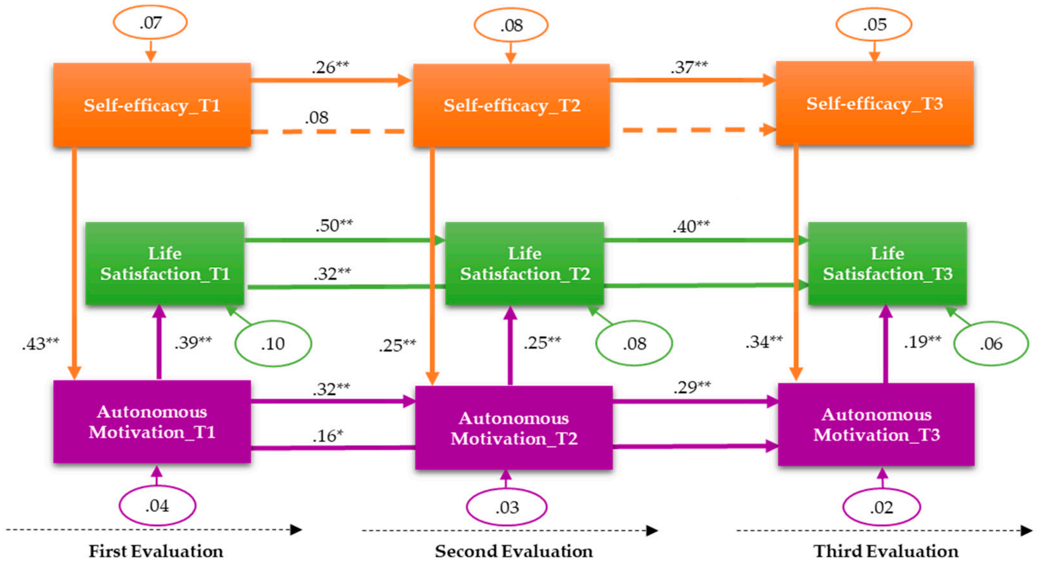

Figure S3. Model 2: Predictive model of life satisfaction in CVD patients considering life satisfaction, autonomous motivation and self-efficacy over time. Standardized coefficients and error terms (circles) are specified. ** $p < .001$; * $p < .05$. Pathway with dashed lines means non-significant relations ($p > .05$).

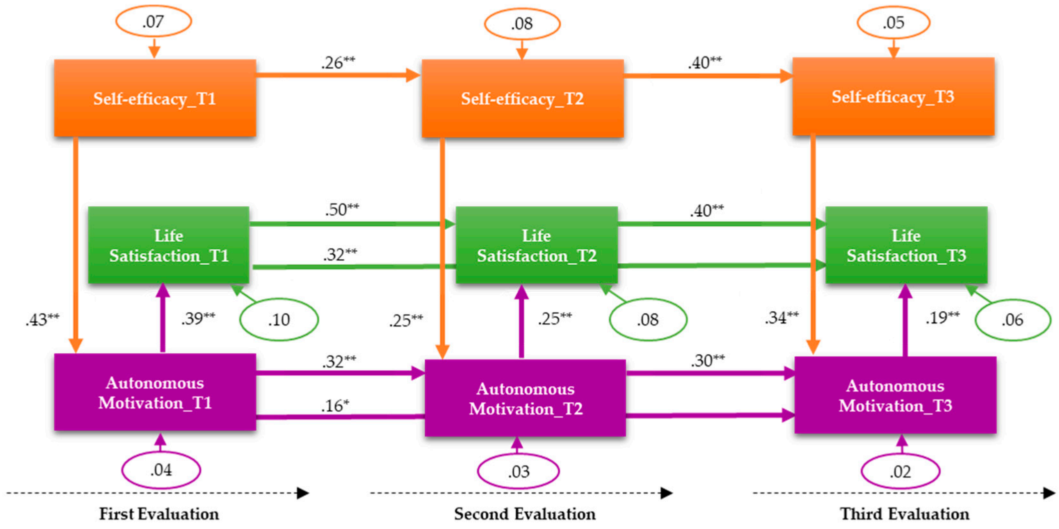

Figure S4. Model 3: Predictive model of life satisfaction in CVD patients considering life satisfaction, autonomous motivation and self-efficacy over time. Standardized coefficients and error terms (circles) are specified. ** $p < .001$; * $p < .05$.

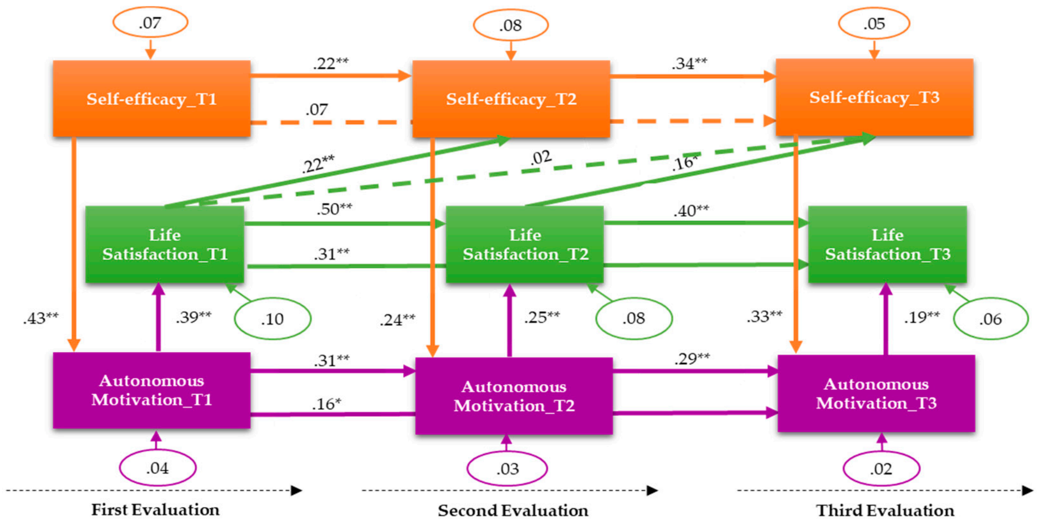

Figure S5. Model 4: Predictive model of life satisfaction in CVD patients considering life satisfaction, autonomous motivation and self-efficacy over time, and the influence of life satisfaction over later measurements of self-efficacy. Standardized coefficients and error terms (circles) are specified. ** $p < .001$; * $p < .05$. Pathways with dashed lines mean non-significant relations ($p > .05$).

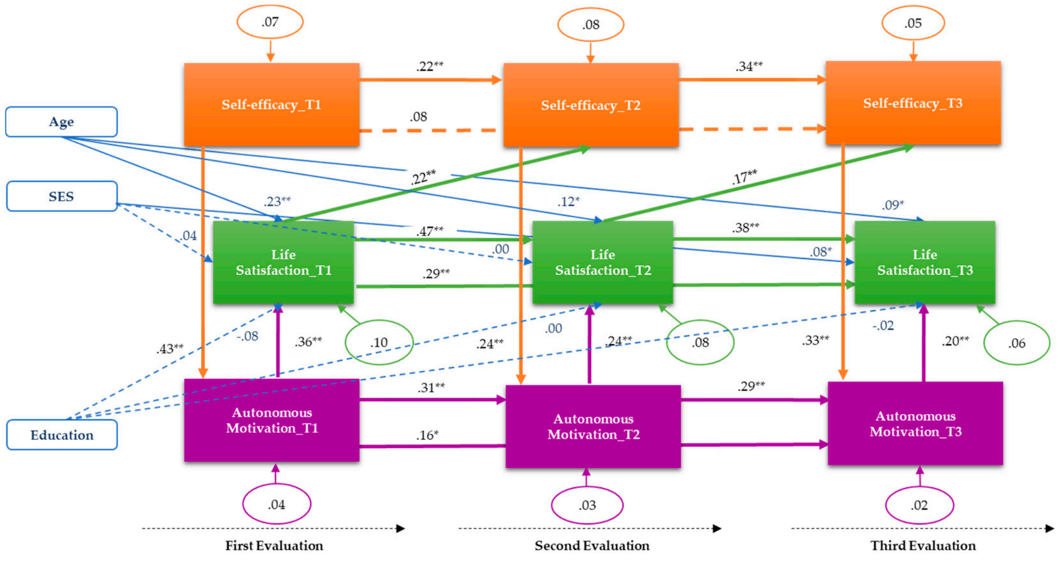

Figure S6. Model 6: Predictive model of life satisfaction in CVD patients considering life satisfaction, autonomous motivation and self-efficacy over time, the influence of life satisfaction over later measurements of self-efficacy, and the influence of sociodemographic variables. Standardized coefficients and error terms (circles) are specified. ** $p < .001$; * $p < .05$. Pathways with dashed lines mean non-significant relations ($p > .05$). SES = Socioeconomic status.

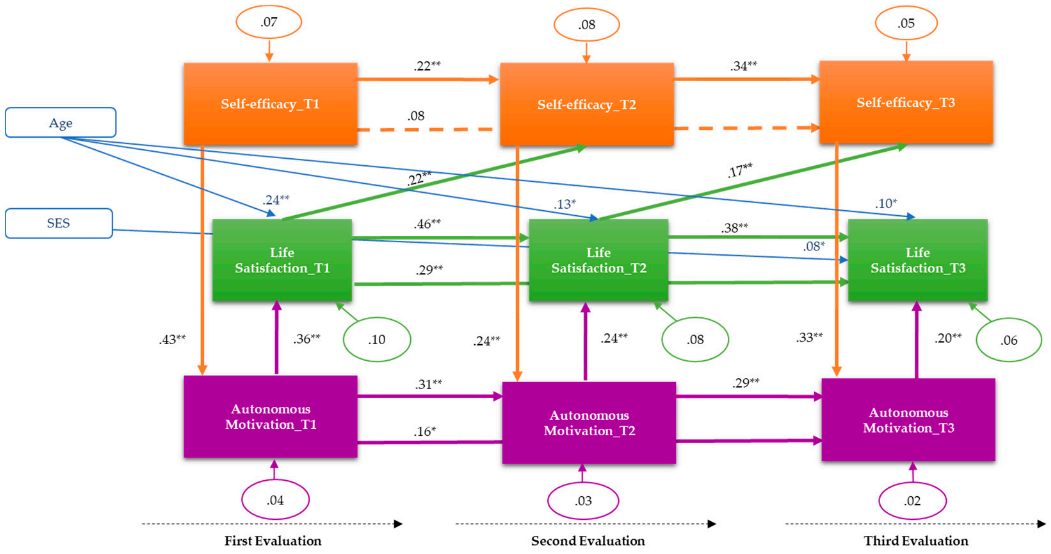

Figure S7. Model 7: Predictive model of life satisfaction in CVD patients considering life satisfaction, autonomous motivation and self-efficacy over time, the influence of life satisfaction over later measurements of self-efficacy, and the influence of sociodemographic variables. Standardized coefficients and error terms (circles) are specified. ** $p < .001$; * $p < .05$. SES = Socioeconomic status.

© 2020 by the authors. Licensee MDPI, Basel, Switzerland. This article is an open access article distributed under the terms and conditions of the Creative Commons Attribution (CC BY) license (http://creativecommons.org/licenses/by/4.0/).
